# Evaluation of a digital game for teaching behavioral aspects of clinical communication in dentistry

**DOI:** 10.1186/s12909-023-04040-7

**Published:** 2023-02-01

**Authors:** Chia-Shu Lin, Cheng-Chieh Yang

**Affiliations:** Department of Dentistry, College of Dentistry, National Yang Ming Chiao Tung University, 155, Sec. 2, Linong St., Taipei, 11221 Taiwan (ROC)

**Keywords:** Gamification, Digital simulation training, Communication, Dentist-patient relations, Dental education

## Abstract

**Introduction:**

Traditionally, dental students learn the skills for dentist-patient interaction and communication via on-site contact with patients, when they start clinical training. However, preclinical students (who have not started clinical practice) have fewer chances to realize the context of dentist-patient interaction. It has remained unclear if a gamification approach via digital media, i.e., a computer role-playing game, can help to learn clinical communication skills. The intervention-based study investigates the effectiveness of the clinical dentist-patient communication (CDPC) game on students’ motivation, beliefs, and self-efficacy to learn behavioral issues of clinical communication.

**Methods:**

Fifty-two dental students (Preclinical group) and 18 dental interns and dentists (Clinical group) played the CDPC game, which consists of 16 scenes of clinical context about dentist-patient communication (less than 40 min for playing), via web browsers. Pre-test and post-test questionnaires were used to assess their motivation, beliefs, and self-efficacy to learn behavioral issues of clinical communication. The effectiveness was examined by comparing pre-test and post-test scores within-subject and between-group difference was compared between Preclinical and Clinical groups, via non-parametric statistical tests.

**Results:**

(A) In the Preclinical group, participants showed a significant increase in motivation and self-efficacy in learning after playing the CDPC game (*p* < 0.05, adjusted of multiple comparison). (B) In contrast, the Clinical group did not show a significant difference before vs. after playing the game. (C) After playing the game, the Preclinical group showed a significant association between motivation and beliefs (*p* = 0.024) and between motivation and self-efficacy (*p* = 0.001); the Clinical group showed a significant association between motivation and beliefs (*p* = 0.033).

**Conclusions:**

The current evidence suggests that gamification of learning helps preclinical students to understand the context of clinical dentist-patient interaction and increase their motivation and self-efficacy to learn behavioral issues of clinical communication.

**Supplementary Information:**

The online version contains supplementary material available at 10.1186/s12909-023-04040-7.

## Introduction

Effective communication and positive relations between doctors and patients play a key role in the improvement of treatment outcomes [1]. Therefore, doctor-patient communication is considered the core element of clinical training for medical/dental students [2, 3]. However, teaching clinical communication skills is challenging because it involves complex interpersonal verbal and non-verbal interactions [4, 5]. Non-verbal interactions, e.g., perceiving patients’ emotional and cognitive status, especially play a critical role in dental treatment. A critical factor for designing the curriculum of clinical communication skills is to establish the environment for students to experience what the verbal and non-verbal interactions are like, i.e., to vividly present the context of dentist-patient interaction [2, 6]. Traditionally, dental students learn the skills of dentist-patient interaction and communication via on-site contact with patients, when they start clinical training, with feedback provided by senior staff [7]. Therefore, in terms of learning communication skills, an active role of patients, such as the notion of patient-centered care, has been highlighted for post-graduate clinical training [8, 9].

Nevertheless, the clinical-based approach to learn communication skills is challenged by three aspects: (A) While younger dentists and the students in their late-year of training (e.g., interns) can realize how dentists interact with patients via clinical practice, it is difficult for preclinical undergraduate (UG) students to gain such an experience. Therefore, the preclinical students may lack the motivation to learn behavioral issues regarding clinical communication because they have not realized what a clinical scenario is like. A vicarious context, such as an interview with simulated patients, is usually integrated with such a course for students to understand the clinical context [10]. (B) Even for the students who started clinical practice, they may have fewer chances to face challenging situations, e.g., to communicate with patients with cognitive deficits or to alleviate fearful patients, because dealing with such difficult patients may lead to greater stress for students [11]. Therefore, the students may not gain much experience in communicating with the patients who require additional skills to interact with. (C) The outbreak of the COVID-19 pandemic has highlighted the role of distant learning in clinical education. The lack of on-site patient contact (partly due to lockdown or quarantine) has greatly changed clinical education [12]. While a lecture on dental anatomy can be effectively delivered by digital approaches, it has remained unclear if students can learn clinical communication distantly via digital approaches.

In terms of the pedagogical methods to present the clinical context of dentist-patient interaction, the use of ‘simulated conditions’ has been reported in the literature [13]. These methods include the use of simulated patients for dental students [10] and role-playing skits for nursing students [14]. The novelty of the current study is to adopt gamification, i.e., a pedagogical approach ‘using game attributes in a non-gaming context’ [15]. In the study, a clinical dentist-patient communication (CDPC) game is designed to present the clinical context of dentist-patient interaction for preclinical students, who have not started dental practice themselves. Furthermore, the CDPC game is designed as a digital game that can be executed via a web browser. This digital game design renders it more approachable for dental students to learn the clinical context of dentist-patient interaction. An earlier randomized controlled trial showed that clinical practice with additional courses on communication skills led to better effectiveness in clinical communication, compared to clinical practice only [16]. Recent studies have revealed that dental students improved in their perception of the quality and safety of healthcare via playing games [17]. It has remained unclear if the digital role-playing game can help students to learn clinical communication skills.

The study investigates the effectiveness of the CDPC game on increasing students’ motivation, beliefs, and self-efficacy to learn behavioral issues of clinical communication. The following hypotheses are tested: (A) Increased learning motivation and self-efficacy are associated with better communication skills in clinical education [18, 19]. Furthermore, clinical communication plays a critical role in the management of dental patients with divergent psychosocial features, such as the patients with fear/anxiety or cognitive deficits [20, 21]. Therefore, we hypothesized that for preclinical students, playing the game would increase their motivation to learn the behavioral issues of clinical communication and their beliefs and self-efficacy in managing patients with different psychosocial background (*Hypothesis 1*). (B) In contrast to preclinical students, clinical dentists have adopted a variety of communication skills for dentist-patient interaction [22]. Therefore, we hypothesized that the effect of the CDPC game on motivation, beliefs, and self-efficacy, would show a significant difference between the preclinical students and participants with clinical experience (*Hypothesis 2*). (C) Learning motivation plays a key role in preclinical dental education, and therefore, we hypothesized that the participants with stronger motivation show higher belief and self-efficacy (*Hypothesis 3*).

## Materials and methods

### Participants and study design

**Fig. 1:**
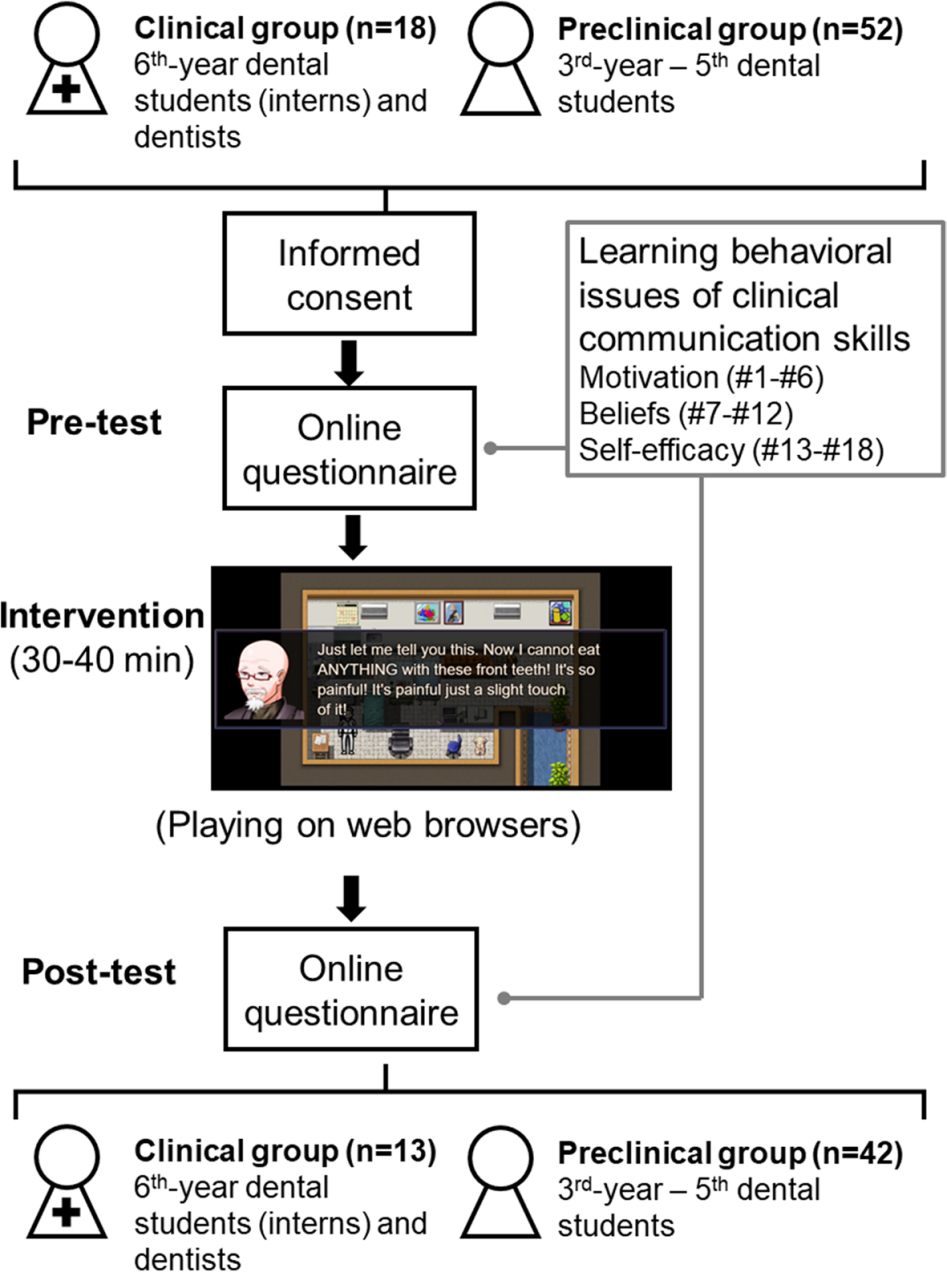
Study design. The study consists of a Preclinical group (undergraduate students without any experience in clinical practice) and a Clinical group (dental interns or dentists who started clinical practice). The participants were asked to finish an online 18-item questionnaire about their motivation, beliefs, and self-efficacy in learning behavioral issues of clinical communication skills (pre-test) and then playing the clinical dentist-patient communication (CDPC) game via a web browser. Subsequently, they were asked to finish the same questionnaire (post-test). 70 subjects (52 Preclinical and 18 Clinical) completed the pre-test questionnaire and among these subjects, 55 (42 Preclinical and 13 Clinical) completed the post-test questionnaire

The study follows a design of non-randomized between-group comparison with a pre-test and a post-test, which was conducted before and after the participants played the CDPC game, respectively (Fig. 1). To test our research hypotheses, two study groups are defined. The Preclinical group consists of the 3^rd^ to 5^th^ year UG students, who have not started clinical training (i.e., to treat dental patients under supervision), according to the guidelines of dental education in Taiwan. The Clinical group consists of the 6^th^-year UG students (i.e., dental interns) and dentists who passed the board examination. Inclusion criteria common to both groups are (1) aged between 20 and 45 years, (2) being able to communicate in Mandarin Chinese and finish the procedure of informed consent independently, and (3) studying at or having graduated from the University. The limitation of the same education background renders the training from dental school balanced between the two groups. Exclusion criteria are (1) having a medical history about addictive behavior of gaming, and (2) feeling emotional disturbance if playing a digital game.

Notably, because the study was conducted during the high time of the COVID-19 pandemic (Nov. 2020 – Jul. 2022), the whole study, including the pre-test and post-test (i.e., completing questionnaires) and intervention (i.e., playing the CDPC game), were conducted online. A digital version of written informed consent was obtained from all participants at the beginning of the study. The study is reviewed and supervised by the Institutional Review Board (IRB) of National Yang-Ming University (IRB code: YM109163E).

### Estimation of sample size

The sample size of the study is estimated based on our primary hypothesis (*Hypothesis 1*) regarding the difference between pre-test and post-test scores in the Preclinical group. Power analysis was performed to estimate the minimal number of participants to reach an effect size of 0.5 (i.e., a medium effect) for Wilcoxon signed-rank test, with Type I and Type II errors controlled at alpha = 0.05 and beta = 0.2, respectively. The analysis was performed using G*Power ver. 3.1.9.4 [23]. Based on the procedure, at least 35 participants are required for the Preclinical group.

### Design of the CDPC game

**Fig. 2:**
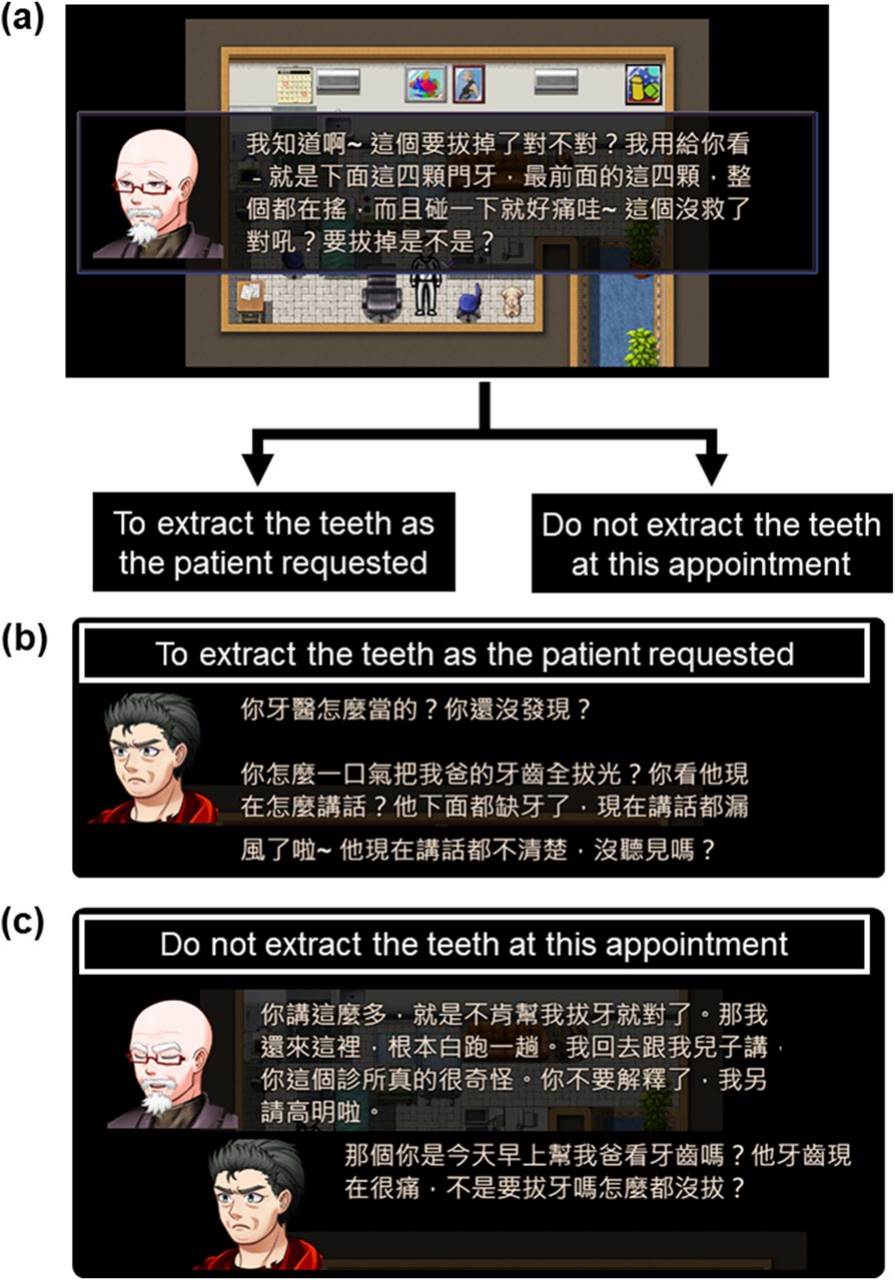
Examples of the game scenes. **a** In Scene #1 (also see Table 1 for a brief description), an old male patient complained that his painful teeth showed severe mobility and cannot eat. The decision may be to extract the teeth or not, and both decisions need to be reached by further discussion with the patients. Poor communication may lead to a worse dentist-patient relationship. In the current scene, the patient is frustrated with the loose teeth and demands them be pulled out. English translation of the original text: “*I know it. The teeth are going to be pulled out, aren’t they? These four front teeth are all loose, and they are very painful when I touch them. They are hopeless to treat and should be pulled out, aren’t they?*” **b** If the player decides to extract the teeth but the dentist fails to inform the potential effect of tooth loss (e.g., feeling difficult to speak), the patient and his family would show great dissatisfaction and argue with the dentist. English translation of the original text: “*How can you just pull out all the teeth from my father? Look at him! How can he speak clearly right now? He cannot speak well because of missing teeth, haven’t you got that?*” **c** If the player decides not to extract the teeth, the reason should be well explained to the patients. Otherwise, the patient and his family may complain to the dentist. English translation of the original text: (The patient) “*Anyway, you just don’t help me with these bad teeth. It’s a waste of my time to come to your clinic. I will talk to my son about this. I’d rather seeing another dentist.*” (The family) “*So you are the dentist who saw my father this morning? He is now extremely painful with the teeth. Why didn’t you pull out them?*”

The CDPC game entitled *Dr. Doog, could you handle this for me?* consists of 16 ‘scenes’ that depict the scenarios when dentists meet dental patients (and sometimes their families) for the first time. The participants play the role of a young dentist (with the gender undefined) working in a local dental clinic, who is accompanied by dental assistants and a personified dog (i.e., *Dr. Doog*), who plays as a funny advisor. In most scenes, participants have two choices to decide how to interact with the patients, which leads to different results (Fig. 2). Notably, *Dr. Doog* may propose some ‘solutions’ according to its observation, which may be correct or just misleading to the participants. The scenes are designed to reflect some clinical scenarios that are challenging for a younger dentist to handle, such as dealing with patients with extreme pain and fear or older patients with different psychosocial background (Table 1). The scenes focus on the context where clinical communication skills are used, such as checking patients’ medical history, explaining a procedure and its risk, and negotiating a treatment plan [6].

**Table 1:** Brief descriptions of the game scenes

#	Scene
1	An elderly man with hearing difficulty, who suffers from severe periodontitis and cannot eat, asks for extracting his front teeth once and for all
2	A car factory worker finds a ‘hole’ in his tooth, which is very sensitive when he drinks water. He sneaks out of work just for fixing the tooth
3	A high school boy with poor oral hygiene comes with his father and asks for ‘cleaning teeth’
4	A young mother brings her child to fix decayed teeth. The child is nervous and unwilling to cooperate. The mother is impatient about this because she needs to go back to the office very soon
5	An undergraduate student has an extremely painful toothache and is very nervous about root canal treatment. Before treatment, the dentist wants to explain everything about the treatment
6	An elderly man who lives alone for many years asks for fixing his old denture, which is actually a broken one that needs a total remake
7	A high school student makes an appointment for fixing teeth, according to the dental assessment in the school
8	A young lady is referred for extracting teeth for subsequent orthodontic treatment. However, the dentist notices that she does not quite get why extraction is necessary for orthodontic treatment
9	A patient appointed for regular dental scaling misses the appointment. He suddenly shows up and asks for treatment when the clinic is closing
10	A patient (who knows the clinic manager very well) asks for refitting his dental bridge, which is actually a temporary bridge. He has worn this temporary bridge for more than half a year
11	The father in #3 considers the dentist as a ‘nice doctor’ and makes an appointment to discuss fabricating a new denture
12	A female truck driver with good oral hygiene asks for a check-up for her toothache. She cannot make a regular appointment because of her busy schedule
13	A granny brings her granddaughter to extract a loose primary incisor. The girl insists to extract the tooth as soon as possible
14	An old woman asks for repairing the wrought wire of her denture. For some reason, she insists on using this unfitting denture
15	A young lady comes with her boyfriend to ‘whiten the front teeth’. She regards it as just a cosmetic procedure. However, the ‘dirty spots’ on her teeth are actually dental caries
16	A patient asks for ‘removing an old dental bridge’ to treat the teeth inside. He does not realize that the removal of the dental bridge means that the bridge will be broken down and a new one needs to make

All the 16 scenes are initially conceived and scripted by the author (C-S Lin) and evaluated by five senior dentists or educators, who have not participated in developing the scenes. The raters evaluated (A) if a scene presents a clinical context related to dental treatment and (B) if the scene helps to bring further discussion between learners and instructors (e.g., senior dental staff). The rating was conducted based on a five-point Likert scale (1: very much disagree, 2: disagree, 3: neutral, 4: agree, and 5: very much agree).

### Production of the CDPC game

The scripted scenes are produced as a digital game by the author (C-S Lin) using the commercial game-making software, RPG Maker MV (DEGICA Co., Ltd., Tokyo, Japan). The RPG Maker series has been used in many studies for producing computer role-playing games for educational purposes, including education of nursing students [24] and physiology and anatomy [25]. To make the CDPC game playable across different platforms, a web-based version is produced so that participants can access the game using either a computer or a mobile phone via a web browser. The Chinese version of the game, which is used in the current study, can be found on the following website: http://thehardproblem.tw/Doog_student/www/game.htm, for non-profit use for educational purposes. For detailed information about the design of game scenarios, please see Supplementary Materials (Table S1).

### Design of the pre-test/post-test questionnaire

The questionnaire for the pre-test and the post-test consists of 18 questions from three domains: Motivation, Beliefs, and Self-efficacy (Table 2), which have been widely investigated in previous studies of clinical communication in medical, dental, and nursing students [14, 18, 19, 26, 27]. The ‘Motivation’ domain consists of six questions that focus on participants’ motivation to learn the behavioral issues of clinical communication, e.g., Question #1 ‘*It is interesting to learn how dentists and patients interact with each other in a clinical context* ‘. The’Beliefs’ domain consists of six questions that focus on participants’ beliefs about the behavioral issues of clinical communication, including their attitudes and thoughts towards dentist-patient interaction, e.g., Question #9 ‘*When dentists discuss treatment plans with older patients, they should keep an eye on the patients’ basic cognitive abilities*’. Finally, the ‘Self-efficacy’ domain consists of six questions that focus on participants’ confidence in understanding and applying the clinical communication skills, e.g., Question #18 ‘*I can spot the association between chronic pain and emotional factors, such as patients’ depression and frustration*’.

**Table 2:** Questions on the motivation, beliefs, and self-efficacy of learning behavioral issues of clinical communications

Domain 1: Motivation	
#1	It is interesting to learn how dentists and patients interact with each other in a clinical context
#2	It is not easy to understand dentist-patient interaction before one starts clinical training
#3	To understand dentist-patient interaction, it is necessary to learn topics in psychology and behavioral science, such as emotion and memory
#4	To understand dentist-patient interaction, it is necessary to learn topics on humanity, such as the association between communication and gender, age, and cultural backgrounds
#5	Learning dentist-patient interaction should be a critical part of the undergraduate curriculum
#6	The topics about dentist-patient interaction do not require additional courses. One can just learn them during clinical practice. (reversed score)
Domain 2: Beliefs	
#7	When patients with chronic orofacial pain complaint about their pain, they often complain deficits of oral functions (e.g., difficulty in eating)
#8	When dentists discuss treatment plans with older patients, psychosocial factors (e.g., treatment is time-consuming) play a key role
#9	When dentists discuss treatment plans with older patients, they should keep an eye on the patients’ basic cognitive abilities
#10	There are very few patients who feel anxiety and fear toward dental treatment. Patients can feel relaxed as long as the environment is comfortable. (reversed score)
#11	Anxious patients may feel uncertain about treatment outcomes and anticipate bad experiences
#12	When dentists discuss a treatment plan with patients, they also need to consider the opinions of family or caretakers
Domain 3: Self-efficacy	
#13	When treating older patients, I can assess if their spatial–temporal orientation and declarative memory are normal
#14	When treating older patients, I can assess their degree of physical frailty
#15	When treating anxious patients, I can differentiate between their trait anxiety and state anxiety
#16	When discussing treatment plans, I understand the individual difference in health beliefs and illness representation between patients
#17	I understand the necessary condition for effective ‘shared decision-making’
#18	I can spot the association between chronic pain and emotional factors, such as patients’ depression and frustration

Participants attended the study by completing the questionnaires and playing the CDPC game online. The questionnaire is produced using Google Form for collecting online responses. The participants are asked to rate how much they agree or disagree with the statement of each question, using a five-point Likert scale (1: very much disagree, 2: disagree, 3: neutral, 4: agree, and 5: very much agree). Upon clicking the webpage of the study, a digital informed consent was displayed and they can decide to join the study or not. If the participants clicked to continue, the pre-test questionnaire would be displayed for them. After completing the pre-test questionnaire, a link to the game was displayed for them to click and play. After finishing the whole game (i.e. completing 16 scenarios), they were required to complete the same questionnaire for the post-test assessment. According our pre-study pilot testing, the duration for completing the whole game (i.e., 16 scenarios) was less than 40 min.

### Statistical analysis

Descriptive data, including the clinical background of the participants and the scores from the questionnaires, are summarized by descriptive statistics (Table 3). Because the questionnaire scores do not follow a normal distribution (Table 4), non-parametric statistical tests are used for testing our major hypotheses. To test *Hypothesis 1*, the Wilcoxon signed-rank test was used to compare the pre-test vs. post-test scores for each question, respectively, for the Pre-clinical and Clinical groups. To test *Hypothesis 2*, the Mann–Whitney U test was used to compare the scores between the Preclinical and the Clinical groups, respectively for each question. To test *Hypothesis 3*, the significant test for Spearman’s correlation coefficient (rho) was used for assessing the association between each pair of the domain, respectively for both groups and pre-test and post-test scores. Within each domain, the scores from all the 6 questions were averaged, and this domain-wise score was used for the analysis of correlation coefficients.

**Table 3:** The demographic features and the clinical experience of the participants

	Sex	After PGY1	PGY1	UG6th	UG5th	UG4th	UG3rd
Pre-test + Post-test	Male	1	2	2	6	1	12
	Female	1	3	4	9	0	12
	Subtotal	2	5	6	15	3*	24
Pre-test only	Male	2	0	0	0	0	0
	Female	1	0	2	1	0	2
	Subtotal	3	0	2	1	7**	2
	Sex	Clinical			Preclinical		
All	Male	7			19		
	Female	11			24		
	Total	18			52		

PGY1: first year of post-graduate training; UG6th, UG5th, UG4th, and UG3rd: undergraduate students at their 6^th^, 5^th^, 4^th^, and 3^rd^ year, respectively

^*^Two subjects in this subgroup did not record their gender

^**^Seven subjects in this subgroup did not record their gender

**Table 4:** Results of descriptive analyses of the question scores

	Pre-test (*n* = 70)						Post-test (*n* = 55)					
	Preclinical			Clinical			Preclinical			Clinical		
	Mn	Md	SD	Mn	Md	SD	Mn	Md	SD	Mn	Md	SD
Motivation												
#1 Interest in learning patient-dentist interaction	4.5	5.0	0.5	4.7	5.0	0.5	4.8	5.0	0.5	4.8	5.0	0.4
#2 Difficulty in understanding patient-dentist interaction	4.4	4.5	0.7	4.3	4.5	0.8	4.5	5.0	0.8	4.5	5.0	0.7
#3 Necessity to learn psychology and behavioral science	4.3	4.0	0.7	4.0	4.0	0.8	4.7	5.0	0.6	4.2	4.0	0.7
#4 Necessity to learn individual background	4.5	5.0	0.5	4.4	4.5	0.7	4.9	5.0	0.4	4.7	5.0	0.5
#5 Inclusion of dentist-patient interaction in curriculum	4.7	5.0	0.6	4.7	5.0	0.5	4.9	5.0	0.4	4.8	5.0	0.4
#6 Learning dentist-patient interaction via practice	3.7	4.0	0.9	3.7	4.0	0.7	4.2	4.0	1.0	4.1	4.0	0.8
Beliefs												
#7 Orofacial pain and deficits of oral functions	4.2	4.0	0.7	4.2	4.0	0.9	4.4	4.5	0.7	4.2	4.0	0.8
#8 Psychosocial factors and treatment plans	4.6	5.0	0.5	4.8	5.0	0.4	4.8	5.0	0.4	4.6	5.0	0.7
#9 Cognitive abilities of elderly patients	4.6	5.0	0.6	4.9	5.0	0.2	4.8	5.0	0.4	4.7	5.0	0.6
#10 Anxiety and fear toward dental treatment	3.8	4.0	1.0	3.9	4.0	0.8	3.7	4.0	1.0	4.0	4.0	0.9
#11 Psychological aspects of anxious patients	4.4	4.0	0.6	4.5	4.5	0.5	4.5	5.0	0.6	4.5	4.0	0.5
#12 Family or caretakers and treatment plans	4.5	4.5	0.5	4.4	4.5	0.6	4.6	5.0	0.5	4.5	5.0	0.5
Self-efficacy												
#13 Assessing orientation and memory	3.3	3.0	0.8	3.8	4.0	0.6	3.8	4.0	0.6	3.8	4.0	0.7
#14 Assessing physical frailty	3.3	3.0	0.7	3.8	4.0	0.8	3.8	4.0	0.8	3.8	4.0	0.8
#15 Differentiating between their trait and state anxiety	2.5	2.0	1.1	2.8	2.5	1.1	3.1	3.0	1.1	3.2	3.0	1.2
#16 Assessing health beliefs and illness representation	3.4	4.0	0.9	3.8	4.0	0.9	4.0	4.0	0.8	4.2	4.0	0.6
#17 Understanding shared decision-making	2.9	3.0	1.0	3.6	4.0	0.9	3.8	4.0	0.8	4.1	4.0	0.6
#18 Assessing emotional factors of chronic pain	3.3	4.0	1.0	3.7	4.0	0.8	3.9	4.0	0.7	4.0	4.0	0.6

*Md* Median, *Mn* Mean, *SD* Standard deviation

Notably, because we hypothesized that difference would be found within each of the question domains (Motivation, Beliefs, and Self-efficacy), adjustment of multiple comparison was performed for each of the domains, which consists of six questions, using Bonferroni correction. Therefore, the alpha value of statistical significance was adjusted to 0.05/6 ≈ 0.008. The effect size (ES) of the difference between pre-test and post-test and that between study groups was estimated using published methods [28]. All the statistical procedures were conducted using IBM SPSS Statistics (ver. 24.0) (IBM, Armonk, NY, USA).

## Results

### Participants

Seventy volunteers were recruited for the current study. Among the participants, 18 are classified as the ‘Clinical’ group, who have started clinical practice, as a 6^th^-year UG student during internship or as a licensed dentist. The other 52 participants are classified as the ‘Preclinical’ group, who have no experience interacting with dental patients. The demographic features and divergence of the clinical experience of the participants are summarized in Table 3. Based on the curriculum of the dental school, the pre-clinical students from the 3^rd^ to 4^th^ year (UG3rd and UG4th, see Table 3) have not attended a compulsory course related to psychology or behavioral science before participating in the study. Though, students may attend some courses regarding humanity and social science, as part of the ‘liberal arts’ modules, during their first year in college. Some of the 5^th^-year students (UG5th) have attended an elective course “Behavioral Dentistry”. All the students have not started clinical practice and gained no experience in patient-dentist communication in a realistic scenario. The association between study groups and sex was not statistically significant (Chi-square test with correction of continuity, *p* = 0.9).

### Evaluation of the game scenarios

Five raters have assessed if the scene helps to bring further discussion between learners and instructors. The raters gave a generally positive rating (average score: 4.7; standard deviation: 0.2) across all the raters. Most scenarios (*n* = 13) received a high (≧4.5) rating from the raters, with the lowest rating (4.3) for question #15 (Table 1). Four of the five raters (except for a teacher of social science) assessed if a scene presents a clinical context related to dental treatment. The raters gave a generally positive rating (average score: 4.7; standard deviation: 0.2) across all the raters. Most scenarios (*n* = 15) received a high (≧4.5) rating from the experts, with the lowest rating (4.2) for question #12 (Table 1).

### Increased motivation to learn, beliefs, and self-efficacy by playing the CDPC game in preclinical students (*Hypothesis 1*)

**Fig. 3:**
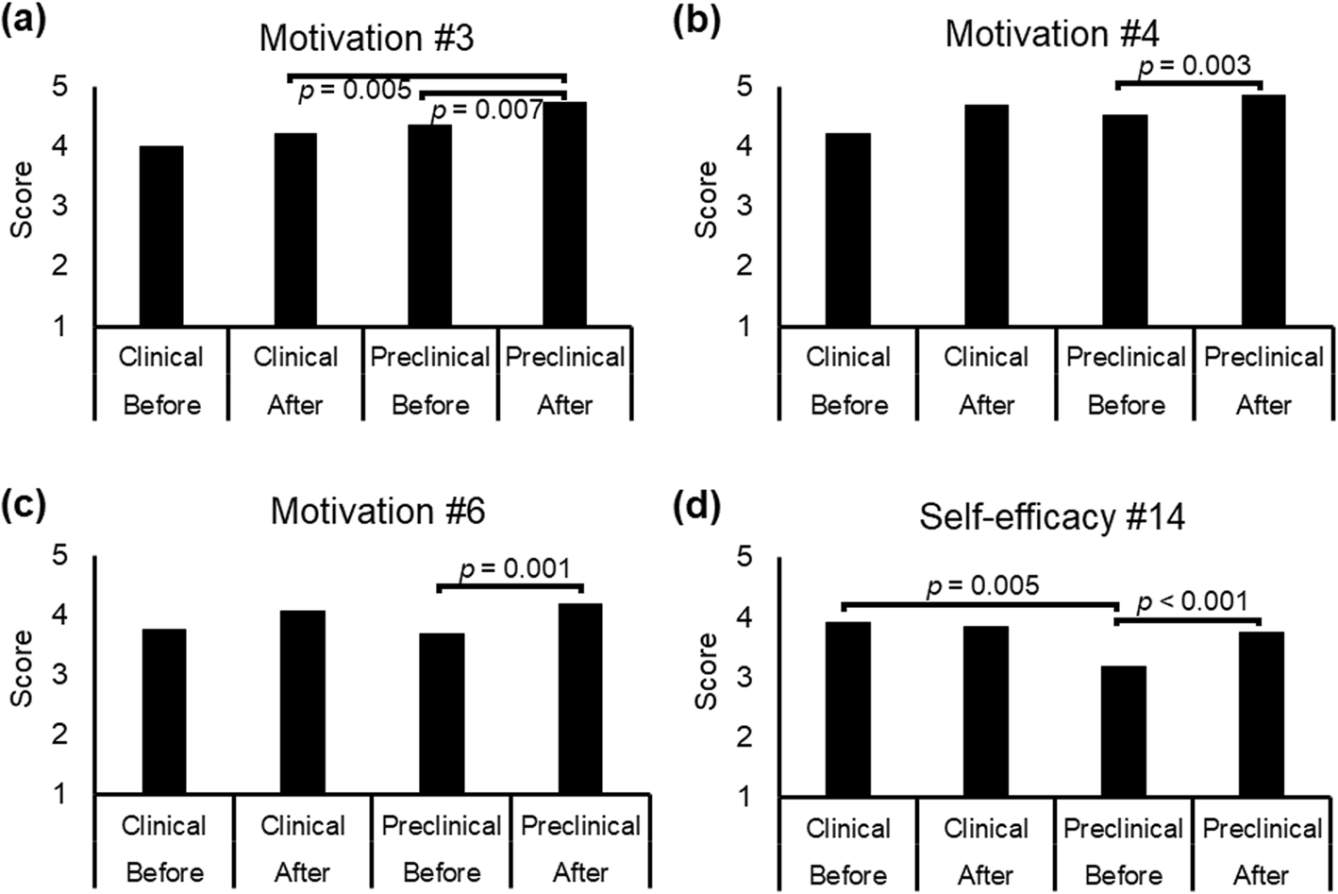
Difference in scores between pre-test vs. post-test and study groups. **a**-**d** In the Preclinical group, three items from the domain Motivation (#3, #4, and #6) and all items from the domain Self-efficacy show significantly increased scores after vs. before playing the game. The difference is not statistically significant in the Clinical group. Between the study groups, the domain Motivation (#3) shows a significant difference after playing the game (panel **a**), and the domain Self-efficacy (#14) shows a significant difference before playing the game (panel **d**)

After vs. before playing the CDPC game, the participants showed a statistically significant increase in Motivation (i.e., Question #3, #4, and #6, Table 5 and Fig. 3a-c) and a statistically significant increase in all questions of Self-efficacy (i.e., Question #13—#18, Table 5). However, the difference in Beliefs was not statistically significant (Table 5). The ES is generally larger in the Self-efficacy domain (0.53 – 0.79, Table 5), compared to the Motivation domain (Table 5). The findings partly support our hypothesis that the CDPC game increased participants’ motivation and self-efficacy, but not beliefs, about learning clinical communication.

**Table 5:** Results of comparison between pre-test vs. post-test and study groups

(A) Pre-test vs. Post-test						
Question	Preclinical (*n* = 42)		Clinical (*n* = 13)		All (*n* = 55)	
	ES	*p*	ES	*p*	ES	*p*
Motivation						
1	-0.39	0.012	-0.16	0.564	-0.34	0.011
2	-0.01	0.955	-0.15	0.589	-0.03	0.837
3	**-0.42**	**0.007**	-0.25	0.366	**-0.38**	**0.005**
4	**-0.46**	**0.003**	-0.59	0.034	**-0.49**	**0.000**
5	-0.21	0.175	-0.16	0.564	-0.20	0.142
6	**-0.50**	**0.001**	-0.45	0.102	**-0.49**	**0.000**
Beliefs						
7	-0.39	0.012	0.00	1.000	-0.30	0.028
8	-0.39	0.012	-0.23	0.414	-0.23	0.088
9	-0.37	0.016	-0.48	0.083	-0.22	0.101
10	-0.05	0.741	0.00	1.000	-0.04	0.767
11	-0.22	0.157	0.00	1.000	-0.16	0.239
12	-0.11	0.467	-0.55	0.046	-0.21	0.127
Self-efficacy						
13	**-0.61**	**0.000**	-0.12	0.655	**-0.52**	**0.000**
14	**-0.59**	**0.000**	-0.12	0.655	**-0.47**	**0.000**
15	**-0.53**	**0.001**	-0.53	0.058	**-0.52**	**0.000**
16	**-0.60**	**0.000**	-0.57	0.038	**-0.59**	**0.000**
17	**-0.79**	**0.000**	-0.57	0.039	**-0.74**	**0.000**
18	**-0.56**	**0.000**	-0.68	0.014	**-0.56**	**0.000**
(B) Preclinical vs. Clinical						
Question	Pre-test (n = 70)		Post-test (n = 55)			
	ES	*p*	ES	*p*		
Motivation						
1	-0.15	0.210	-0.01	0.934		
2	-0.04	0.719	-0.10	0.451		
3	-0.16	0.186	**-0.37**	**0.005**		
4	-0.08	0.486	-0.18	0.182		
5	-0.07	0.562	-0.10	0.458		
6	-0.06	0.606	-0.12	0.364		
Beliefs						
7	-0.02	0.874	-0.10	0.460		
8	-0.23	0.050	-0.11	0.421		
9	-0.28	0.017	-0.06	0.669		
10	-0.05	0.697	-0.12	0.360		
11	-0.03	0.783	-0.08	0.546		
12	-0.02	0.896	-0.03	0.836		
Self-efficacy						
13	-0.26	0.028	-0.08	0.572		
14	**-0.33**	**0.005**	-0.04	0.750		
15	-0.10	0.422	-0.06	0.674		
16	-0.21	0.084	-0.07	0.626		
17	-0.30	0.011	-0.16	0.229		
18	-0.13	0.261	-0.04	0.747		

*ES* Effect size. The bold type indicates the value that is statistically significant (after correction of multiple comparison)

### Difference between the preclinical students and participants with clinical experience (*Hypothesis 2*)

Before playing the CDPC game, the score was significantly higher in the Clinical group (mean ± standard deviation: 3.8 ± 0.8), compared to the Preclinical group (3.3 ± 0.7), for Question #14 of Self-efficacy (Mann–Whitney U test, two-tailed *p* = 0.005) (Table 5 and Fig. 3d). After playing the CDPC game, the score was significantly lower in the Clinical group (4.2 ± 0.7), compared to the Preclinical group (4.7 ± 0.6), for Question #3 of Motivation (Mann–Whitney U test, two-tailed *p* = 0.005) (Table 5 and Fig. 3a).

### Association between each domain (*Hypothesis 3*)

**Fig. 4:**
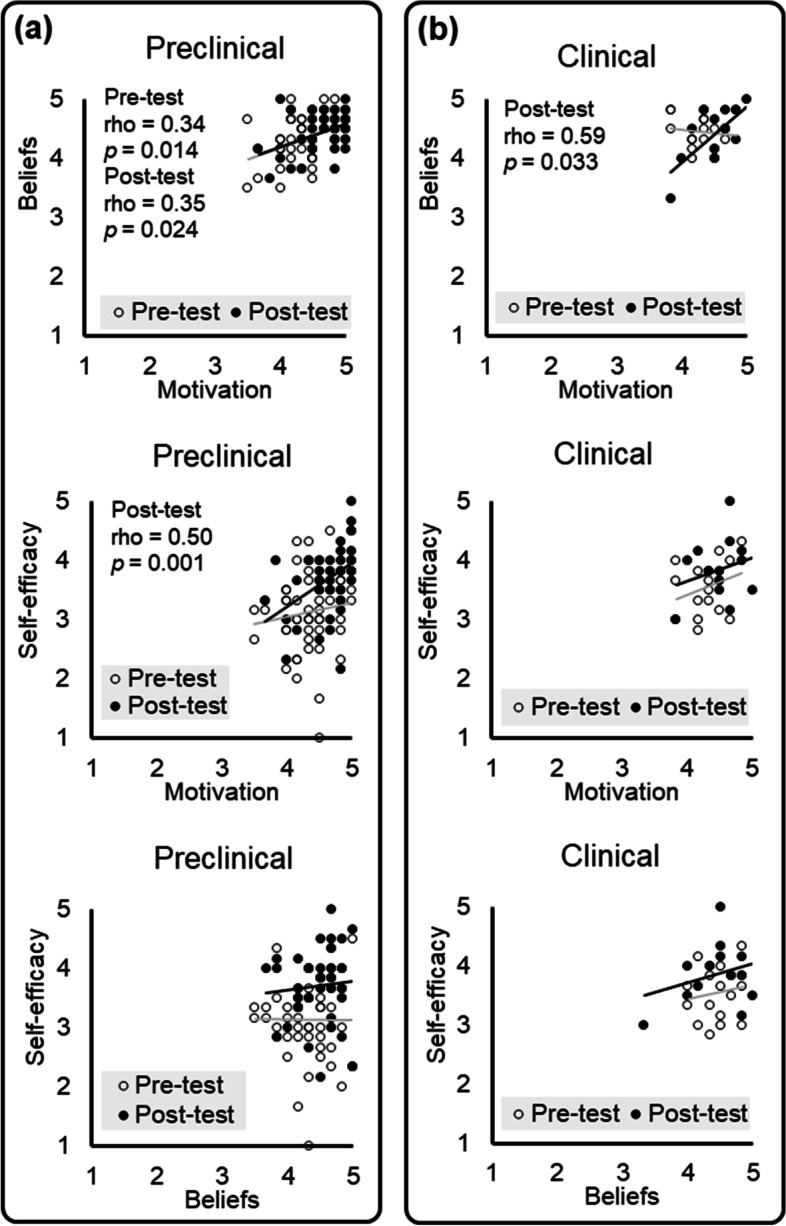
The association between the domains in different study groups. **a** In the Preclinical group, the score of Motivation is significantly correlated with the score of Beliefs before and after playing the game as well as the score of Self-efficacy after playing the game. **b** In the Clinical group, the score of Motivation is significantly correlated with the score of Beliefs after playing the game

The correlation of the score between each domain before (i.e., pre-test) and after (i.e., post-test) playing game is shown in Fig. 4, respectively for the Preclinical (Fig. 4a) and the Clinical (Fig. 4b) groups. Before playing the game, a statistically significant correlation was found only in the Preclinical group between Motivation and Beliefs (rho = 0.34, two-tailed *p* = 0.014, Fig. 4a). This positive correlation remained significant after the participants played the game (rho = 0.35, two-tailed *p* = 0.024, Fig. 4a). Furthermore, after playing game, Motivation and Self-efficacy became significantly correlated in the Preclinical group (rho = 0.50, two-tailed *p* = 0.001, Fig. 4a) and Motivation and Beliefs became significantly correlated in the Clinical group (rho = 0.59, two-tailed *p* = 0.033, Fig. 4b).

## Discussion

### Summary of the major findings

In the current study, we investigated the effectiveness of playing the CDPC game on the motivation, beliefs, and self-efficacy of learning behavioral issues of clinical communication, respectively for participants with (Clinical) and without (Preclinical) clinical experience. Our major findings include: (A) In the Preclinical group, participants showed a significant increase in motivation and self-efficacy of learning after playing the CDPC game (Table 5 and Fig. 3). (B) In contrast, the Clinical group did not show a significant difference before vs. after playing the game. Before playing the game, the Clinical group showed a higher self-efficacy, compared to the Preclinical group (Table 5 and Fig. 3). (C) After playing the game, the Preclinical group showed a higher association between motivation and self-efficacy; the Clinical group showed a higher association between motivation and beliefs (Fig. 4), in terms of learning the behavioral issues of clinical communication.

### The effectiveness of the CDPC game on learning behavioral issues

After vs. before playing the game, the preclinical students showed a higher motivation to learn by agreeing that ‘*To understand dentist-patient interaction, it is necessary to learn topics on psychology and behavioural science, such as emotion and memory*’ (#3) and ‘*To understand dentist-patient interaction, it is necessary to learn topics on humanity, such as the association between communication and gender, age, and cultural backgrounds*’ (#4). The findings correspond to the design of the scenes of the CDPG, which emphasized the role of psychological factors in dentist-patient communication. For example, the scenes #5, #9, and #11 describe the behavior of patients with stronger fear and anxiety toward dental treatment, and the scenes #1, #6, and #14 focus on the geriatric patients who may be difficult to communicate due to memory deficits. The increased motivation of learning may be associated with the use of realistic scenario of patient-dentist communication, as described in the game scenarios. Consistent to our findings, in nursing students, learning with realistic cases of patient-nurse communication was associated with greater learning motivation, compared to a lecture-based learning [18]. In dental students, a combination of lecture and case-based courses increased their motivation of clinical practice [29]. Importantly, the focus on age-specific issues and psycho-socio-cultural diversity of patients echoes the key components of the medical curriculum for clinical communication [2]. After playing the game, the Preclinical students also showed a stronger disagreement that ‘*The topics about dentist-patient interaction do not require additional courses. One can just learn them during clinical practice*’ (#6). The finding is interesting because, in the conventional dental curriculum, behavioral issues such as communication skills are mainly taught when students start clinical training in the latter years. The findings are consistent with previous results, which showed that UG learners felt more interested and self-confident in clinical communication, after attending a communication course [10].

It is noteworthy that after playing the game, the preclinical students showed an overall higher self-efficacy in the behavioral issues of clinical communication skills (i.e., #13—#18 for Self-efficacy). It should be noted that the preclinical students did not fully receive a lecture or practice about these issues. For example, the term ‘declarative memory’, ‘physical frailty’, or ‘shared decision-making’ were not taught to a 3^rd^-year UG dental student. Therefore, it is not surprising that they show a low score for these before playing the game (Fig. 3d). Interestingly, after playing the game, the students increased their self-efficacy to a level similar to the Clinical participants (Fig. 3d). According to Social Cognitive Theory, such an increased self-efficacy may reflect higher expectancies of the outcome of clinical communication [30]. For example, after playing scenes #1, #6, and #14, which relate to the management of older patients, students become aware that understanding patients’ physical conditions would be critical to oral healthcare of older patients, which echoed their scores for Question #14 of Self-efficacy (Fig. 3d).

### Differential effects of the game on participants with vs. without clinical experience

In contrast to the Preclinical group, the Clinical participants did not show a significant difference in any of the domains after vs. before playing the game. A potential explanation for the lack of significant difference is that in the Clinical groups, the participants have already gained a high score in these domains before playing the game (Table 4). This may reflect the fact that they have experienced how dentists and patients interact during clinical training. For example, after playing the game, the Preclinical participants showed a higher score of Self-efficacy in Question #13 ‘*When treating older patients, I can assess if their spatial–temporal orientation and declarative memory are norma*l’ (from 3.3 ± 0.8 to 3.8 ± 0.6). In contrast, in Clinical participants, playing the game did not lead to a change in the score of the same question (from 3.8 ± 0.6 to 3.8 ± 0.7) because they have already recognized that memory assessment is critical to managing older patients, even before playing the game. Critically, the findings suggest that to the Preclinical students who have not contacted with patients, playing the game would help them gain experience in the context of clinical communication, which dentists would face in their career.

### Association between each domain of learning

Previous studies suggested that dental students’ health belief attitudes improved via a training of cross-cultural communication [27]. Nursing students showed improved beliefs and self-efficacy in clinical communication, when receiving virtual simulation of clinical scenarios [26]. The findings suggest the benefits of case-based learning on changing the beliefs of students in health professions. In our study, the pre-test vs. post-test and the between-group analyses did not show a significant difference in the Beliefs domain. The findings may echo the nature of the CDPC game, i.e., to present realistic clinical scenarios (of the patient-dentist communication) to pre-clinical students. The question items for this domain highlighted the importance of health beliefs in communication, e.g., Question #7 (the role of oral function in orofacial pain), #8 (the role of psychosocial factors for older patients), #11 (the importance of emotional factors), and #12 (the communication with patients’ family) (Table 2). Instead, students are not expected to self-learn the knowledge regarding these beliefs – which would be further taught and discussed in the class. The score from this domain is positively correlated with the score from the Motivation domain in the Preclinical group (for both pre-test and post-test) and the Clinical group (for post-test only) (Fig. 4). Before playing the game, the preclinical students who showed a higher motivation to learn the behavioral issues may acquire the relevant knowledge from other media. For example, the students who realize the importance of patients’ divergence in psychosocial factors (as indicated in Question #4) would be more enthusiastic about learning the knowledge about the healthcare of dementia or mental illness from the Internet or books, and therefore, they would show stronger beliefs that individual cognitive and emotional status should be evaluated (as indicated in Question #9 and #10). After playing the game, the students with a higher learning motivation showed both stronger beliefs and higher self-efficacy. The positive correlation between motivation and self-efficacy suggests that the students got familiar with the clinical context, where the ability to manage patients with divergent psychosocial background is important to dentist-patient interaction.

In contrast to the Preclinical group, the Clinical group only showed a significant correlation in the scores between Motivation and Beliefs after playing the game. After playing the game, the interns and dentists may feel a stronger need to revisit their skills of clinical communication, when the game scenes echoed their own experience of treating patients, i.e. the approaches that they used for communication. It is noteworthy that although participants in this group have much experience in interacting with patients, that does not necessarily mean the participants can fully master the communication skills. An earlier survey in the United States showed that the diversity of communication techniques used by dentists was low [22]. Therefore, the clinical participants may become aware of the different approaches to communication skills after they play the game.

### Limitation of the study

The findings in the study should be interpreted carefully with the following limitations. First, though power analysis revealed that the sample size of the study meets the criteria for evaluating our primary hypothesis (i.e., *Hypothesis* 1), it is limited to more sophisticated statistical models, such as a multivariate analysis. Therefore, the associations between the intervention and the variables were not fully explored. Second, because the study was conducted fully online during the COVID-19 pandemic, some qualitative information, which need to be collected via a face-to-face interview, was not recorded. For example, the participants in the Clinical group may have different interpretations of some scenes presented in the game, based on their own experience of clinical practice. How their prior experience of clinical practice affects their understanding of the game scenes would be a critical issue to explore in future research. Third, our findings do not suggest that the preclinical students who showed greater changes via playing the game would perform better in clinical communication when they start clinical training. Instead, our findings suggest the potential use of the digital game, as a media for presenting clinical scenarios for students, in a lecture-based class. Further investigation, especially a longitudinal study that follows the students’ performance from preclinical to clinical stages of learning, would help to clarify the association between the learning motivation and self-efficacy and clinical performance of clinical communication.

## Conclusion

The current evidence suggests that playing the CDPC game, which helps preclinical students to understand the context of clinical dentist-patient interaction, would increase their motivation and self-efficacy to learn behavioral issues of clinical communication.

## Supplementary Information

**Additional file 1:** **Table S1.** Description and design of the game scenarios. Please note that the scenarios are designed based on the clinical context of a local clinic in Taiwan. Therefore, the treatment procedures, the patient-doctor relationship, and the health-related policies may differ from those in other countries.

## Data Availability

The datasets generated during and analyzed during the current study are not publicly available due to regulations on the privacy of the subjects according to the guidelines from the local Internal Review Board but are available from the corresponding author on reasonable request.
