# Supplementary material for: Evaluation of a digital game for teaching behavioral aspects of clinical communication in dentistry
Source: BMC Med Educ. 2023 Feb 1;23:78. doi: 10.1186/s12909-023-04040-7 (PMC9889244; doi:10.1186/s12909-023-04040-7)
Supplement: Supplementary file 1 — Additional file 1: Table S1. Description and design of the game scenarios. Please note that the scenarios are designed based on the clinical context of a local clinic in Taiwan. Therefore, the treatment procedures, the patient-doctor relationship, and the health-related policies may differ from those in other countries. [file 12909_2023_4040_MOESM1_ESM.docx]

**Table 1** Description and design of the game scenarios. Please note that the scenarios are designed based on the clinical context of a local clinic in Taiwan. Therefore, the treatment procedures, the patient-doctor relationship, and the health-related policies may differ from those in other countries.

| ID | Scenario description | Choices | Story branching | Study purpose (for students) | Themes for discussion (for teachers) |
| --- | --- | --- | --- | --- | --- |
| 1 | 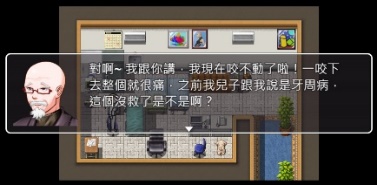 An elderly man with hearing difficulty, who suffers from severe periodontitis and cannot eat, asks for extracting his front teeth once and for all. | **[A]** You extracted the teeth as the patient requested. | **[A]** The patient cannot speak (pronounce) well because of the missing front teeth. His family was unhappy with this and argued it with you. | • Understanding the importance of functional aspects of making clinical decisions  • Recognizing the interpersonal stress in the patient-dentist relationship  • Differentiating a medical decision and the process (communication) to achieve that decision. | • What are the most critical oral functions for elderly people (eating? speech? esthetics?)  • What are the points to be considered when dentists talk about the side effect of treatment?  • When patients or family make a complaint, how should we improve the patient-dentist relationship? |
|  |  | **[B]** You did not extract the teeth at this appointment. | **[B]** The patient still felt very painful and cannot eat. His family was unhappy with this and argued it with you. |  |  |
| 2 | 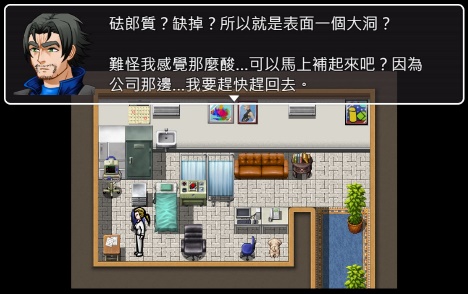 A very busy mechanic walked in and asked for ‘filling a hole’ in his tooth. The tooth is very sensitive even when he drinks. | **[A]** You Identified the ‘hole’ as a result of cervical abrasion and restored it. | **[A]** After restoration, the patient still felt very painful with spontaneous pain. | • Understanding the social factors related to patients’ behavior during dental visiting  • Identifying the priority of treatment for painful patients | • When a patient with very busy work comes to see dentists (without an appointment), what would be the potential reasons?  • The patient thought there was a ‘hole’ but his impression may be wrong. How should we clarify the wrong impression to him?  • If the patient has not much time for full examination/treatment, how should we negotiate with the patient with a treatment plan? |
|  |  | **[B]** You suspected the case was pulpitis and asked him for more examination. | **[B]** The patient refused more examination because he needed to get back to work soon. |  |  |
| 3 | 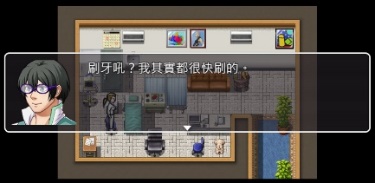A father brought his son for dental scaling just before the closing hour. The boy, however, has very poor oral hygiene. | **[A]** You conduct dental scaling and thorough oral hygiene instruction to the patient and his family. | **[A]** The father is much impressed by the dentist and made an appointment for himself. However, the OHI took much time and violated the break time for dental assistants. | • Understanding the psychosocial factors related to oral hygiene  • Recognizing the importance of team working | • For patients with poor oral hygiene, what are the underlying psychosocial factors?  • Does oral hygiene instruction help build up the patient-dentist relationship?  • Should the break time of the team be delayed, if it’s necessary to spend more time on patients? Is there a way to keep both balanced? |
|  |  | **[B]** You conduct dental scaling only. | **[B]** The father is satisfied with the dentist. |  |  |
| 4 | 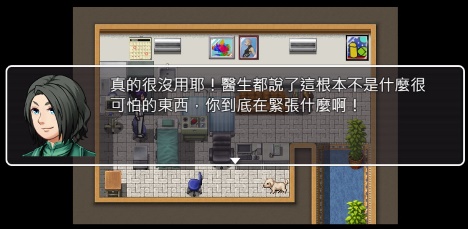  A mother brought her kid for filling caries. The kid was much nervous about it and unwilling to open his mouth. The mother was so frustrated because she still had lots of work to do and can’t spend more time on this. | **[A]** You found many dental caries and filled them all at once. | **[A]** The kid had all caries fixed but the duration of treatment was prolonged. The mother was frustrated. | • Recognizing the importance to balance between ‘time factor’ and treatment goals  • Understanding the role of communication in making an appointment for children and their family | • If child patients cannot cooperate well, what points should we communicate with their parents?  • When we form a treatment plan for child patients, why should take their parents’ points into consideration? |
|  |  | **[B]** You managed some dental caries and filled the others in the next appointment. | **[B]** The kid had some caries fixed. The mother was worried about the next appointment (if the kid does not cooperate well). |  |  |
| 5 | 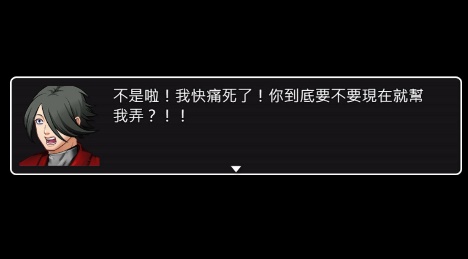A college student made an appointment because severe toothache. He was very anxious and expected the dentist would ‘kill the dental nerve’ (i.e., endodontic treatment). | **[A]** You have explained about the pulpitis and endodontic treatment, and performed an emergent treatment for alleviating pain. | **[A]** The pain was alleviated but not fully relieved. | • Recognizing what the dentist (in this scenario) did wrong in communication.  • Understanding the role of emergent treatment and the importance to alleviate patients’ major symptoms. | • When there are multiple points to talk to patients (e.g. the complexity of endodontic treatment), how would you prioritize these points?  • How to differentiate between a temporary (emergent) procedure and full treatment to patients?  • Is it possible that the patients who are too painful/anxious to attend to dentists’ instruction? |
|  |  | **[B]** You took much explained (with very much detail) the pulpitis and endodontic treatment. You asked the patient to come next time for a full-set treatment. | **[B]** The patient felt frustrated that the dentist did not conduct any treatment, even a temporary one. |  |  |
| 6 | 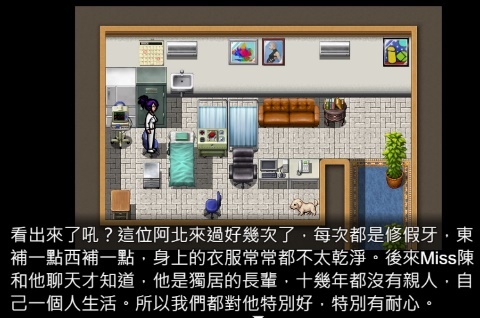 An elderly man who lives alone for many years asks for fixing his old denture, which is actually a broken one that needs a total remake. | **[A]** You fixed the broken denture temporarily and spent much time persuading the patient to come back for a remake. | **[A]** The patient felt unhappy with the dentist’s persuasion of remaking a new denture. | • Understanding the dilemma between an ‘optimal solution’ (i.e., remaking a new denture) and a ‘satisfying solution’ – how patients react to each of them  • Understanding the psychosocial background of an elderly person living alone. | • The patient thought the broken denture can be ‘fixed’ just like a new one. How should we clarify this misconception to him?  • When a remake is needed, how should we tell patients the necessity to do it?  • Is it possible that the patient relucted to remake a new denture because of his psychosocial background? |
|  |  | **[B]** You fixed the broken denture temporarily, without persuading him to remake it. | **[B]** The patient gave his thanks to the dentist. |  |  |
| 7 | 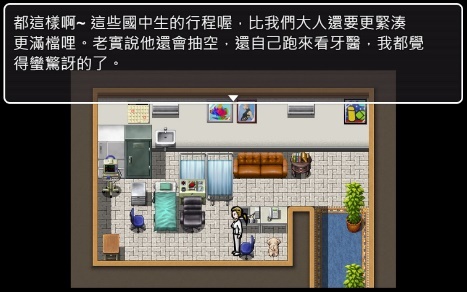 A high school student makes an appointment for fixing teeth, according to the dental assessment in the school. | **[A]** You fixed just dental caries according to the assessment from the school. | **[A]** The student had the assessment form checked and left. | • Recognizing the motivation for a dental check-up.  • Understanding the fact that sometimes, parents themselves do not understand well the oral hygiene of their children. | • What if a patient comes to you asking for ‘minimal treatment’?  • Is it possible that parents themselves do not understand the oral hygiene of their children? What if you did the right decision – but it was misunderstood by the parents? |
|  |  | **[B]** You found more caries and fixed all of them. | **[B]** The student had the assessment form checked and left. Later, his parent called and complained about why the dentist fixed more teeth than what the assessment said. |  |  |
| 8 | 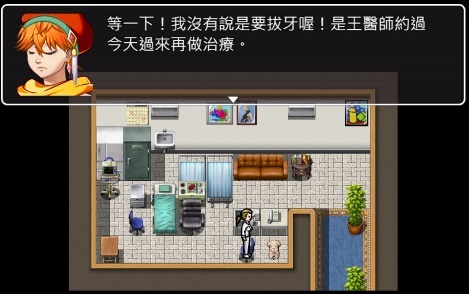 A young lady is referred for extracting teeth for subsequent orthodontic treatment. However, the dentist notices that she does not quite get why extraction is necessary for orthodontic treatment. | **[A]** You persuaded the patient to extract the teeth and completed dental scaling. | **[A]** The patient had her teeth extracted and other teeth cleaned, feeling satisfied with the treatment. | • Understanding the importance of cross-disciplinary teamwork in dental treatment.  • Differentiating between the major aims of a treatment and the additional benefits of a treatment. | • When patients have problems with the major aim of treatment (here, the reason for extraction for orthodontic treatment), how should we further communicate with them?  • Should we make the medical decisions ourselves with patients? Or should the decisions be left to the specialists (e.g., orthodontists)  • Is that a good strategy to satisfy patients by providing additional service (e.g., scaling) that is not part of the original treatment plan? |
|  |  | **[B]** You persuaded the patient to extract the teeth. | **[B]** The patient had her teeth extracted. |  |  |
| 9 | 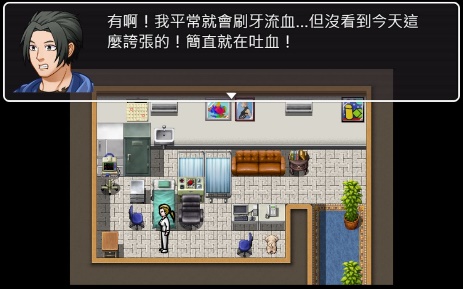A patient appointed for regular dental scaling misses the appointment. He suddenly shows up and asks for treatment when the clinic is closing. | **[A]** You had the patient come next time for a new appointment (since he was late). | **[A]** The patient just left. | • Understanding that patients delay or miss an appointment is common in clinics. Communication skills are critical in negotiating with patients.  • Understanding the importance of precaution against any discomfort during treatment (e.g., bleeding), even for a simple and regular procedure.  • Recognizing that patients who frequently delay or miss an appointment may feel highly anxious about dental treatment. | • If patients delay or miss an appointment, what psychosocial factors should we pay attention to? (e.g. are patients too nervous to visit dentists? Are they too busy to keep the schedule?)  • Is that possible that patients hold a wrong impression (or beliefs) regarding pain and bleeding – they misconceive that all the discomfort is caused by dentists. How should we clarify this with them? |
|  |  | **[B]** You tried to finish the case (scaling) before the clinic is closing. | **[B]** The patient was shocked by severe bleeding during scaling and misrecognized the bleeding as a result of the dentist’s problem. |  |  |
| 10 | 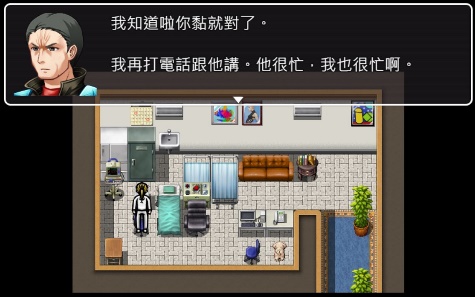  A patient (who knows the clinic manager very well) asks for refitting his dental bridge, which is actually a temporary bridge. He has worn this temporary bridge for more than half a year. | No choice available in this scenario | No branching story available for this scenario | • Understanding that some patients would ‘distort’ the patient-dentist relationship for their ‘special roles’ (e.g., a VIP of the clinic manager) | • If patients consider themselves a VIP and ask for special ‘care’ (which is unnecessary), how should we communicate with them?  • What if the so-called ‘VIP’ patients just have a wrong belief in their oral health? (e.g., treating a temporary device as a permanent one)? What strategies can we use to help them? |
| 11 | 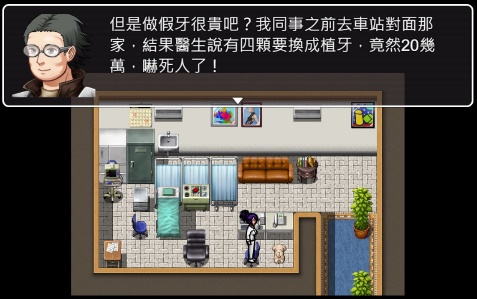The father in Scenario #3 regards the dentist as a ‘nice doctor’ and makes an appointment to discuss fabricating a new denture. | **[A]** You first discussed with the patient the cost of fabricating a denture – which the patient really cares about. | **[A]** The patient made a new appointment coming next time. | • Understanding that some patients are worried about the cost of a treatment (e.g., fees) more than its medical effectiveness.  • Understanding that some patients may not trust dental personnel (e.g., thinking that dentists charge a higher fee) | • What if patients do not trust your capability and judgment, simply because you are not a senior staff? Any good strategy to improve this relationship?  • Is that a good approach to improving the patient-dentist relationship by having a ‘bargain’ with them (e.g., having a discount for patients)?  • When patients over-emphasize the cost of treatment, how should we redirect the topic to the treatment procedure per se? |
|  |  | **[B]** You first examined the patient’s oral condition and took an X-ray film. | **[B]** The patient made a new appointment coming next time. |  |  |
| 12 | 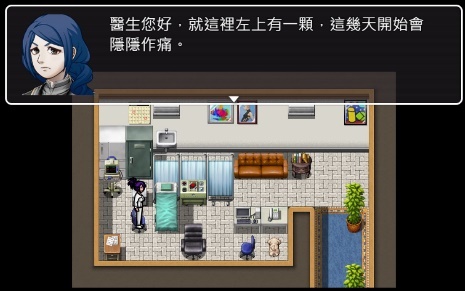 A female truck driver with good oral hygiene asks for a check-up for her toothache. She cannot make a regular appointment because of her busy schedule. | **[A]** You expressed your surprise that a truck driver has such good oral health. | **[A]** The patient argued with the dentist for the severe bias on the link between oral health and occupations. | • The scenario aims to impress dental students on how social biases will severely harm the patient-dentist relationship.  • Understanding that in any condition, dentists should NOT have social, occupational, or gender bias influence their medical judgment. | • We know that social biases will harm the patient-dentist relationship. How can we avoid such biases during communication? |
|  |  | **[B]** You expressed your surprise that a truck driver is a woman. | **[B]** The patient argued with the dentist for the severe bias on the link between occupations and gender. |  |  |
| 13 | 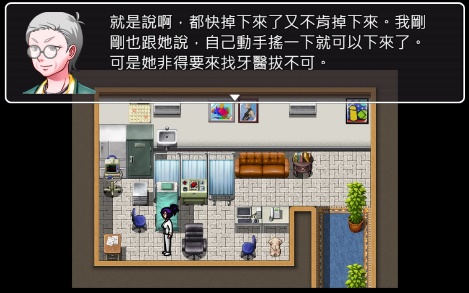A granny brings her granddaughter to extract a loose primary incisor. The girl insists to extract the tooth as soon as possible. | **[A]** You thought that extraction is not necessary and asked the patient back home and have it spontaneously exfoliated. | **[A]** The granny agreed with you and hoped the tooth to exfoliate spontaneously. | • Understanding that both biological (e.g., root resorption) and psychosocial factors are critical to medical decisions.  • Recognizing if the family have any misconception about oral health, e.g., considering primary teeth as ‘unimportant’ or anesthesia as unnecessary | • In this scenario, the kid has her ‘special reason’ to extract the tooth. Should we listen to her opinion or just follow her granny’s opinion?  • Why does the granny underestimate the importance of primary teeth? Can you figure out any reason (e.g., for her own experience as a kid, or for the message from the media)? |
|  |  | **[B]** You extracted the primary tooth with local anesthesia. | **[B]** The granny felt more confused about why extraction and anesthesia were needed. |  |  |
| 14 | 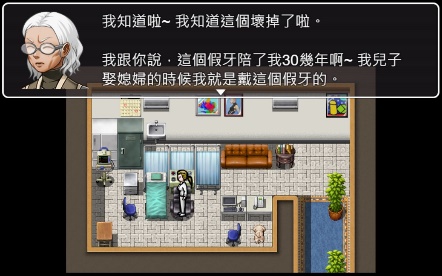 An old woman asks for repairing the wrought wire of her denture. For some reason, she insists on using this unfitting denture. | **[A]** You warned the patient that more repair will damage the denture and asked her to remake a new one. | **[A]** The patient was reluctant to give up the broken denture because of its special meaning to her. She thought the dentist is just a zealous vendor. | • Understanding the importance of assessing the cognitive abilities of elderly patients.  • Understanding the importance of assessing the emotional experience of elderly patients. | • When elderly patients insist on their own opinions, is it possible that they cannot understand the pros/cons of treatment due to lower cognitive abilities (e.g., failure to attend to dentists’ instruction)?  • Also, is that possible that elderly patients have a special emotional attachment to something (e.g., keeping the denture as a memorial)? How should we communicate this with patients? |
|  |  | **[B]** You tried to fix it as possible as you can. However, it won’t improve much. | **[B]** The patient felt satisfied that the denture was ‘fixed up’. However, it will break again in a short time. |  |  |
| 15 | 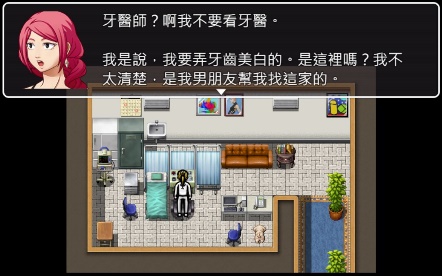 A young lady comes with her boyfriend to ‘whiten the front teeth’. She regards it as just a cosmetic procedure. However, the ‘dirty spots’ on her teeth are actually dental caries. | **[A]** You told the patient that the ‘dirty spots’ are dental caries and after restoration, which would become prettier. | **[A]** The patient focused on the removal of the ‘dirty spots’ and felt it was prettier. | • Understanding that patients’ priority of treatment may be different from dentists’ perspective  • Understanding the importance to clarify the rationale for treatment to patients (e.g. for cosmetic reasons or for curing a disease)  • Recognizing the role of significant others (e.g., the boyfriend) in making medical decision | • The patient’s beliefs and cognition about health and diseases are different from the dentist’s perspective. How should we help her to reshape her beliefs and cognition?  • If the patient just cares about cosmetic factors, should we just focus on that part first?  • In this scenario, her boyfriend thought the restoration is good enough. How should we evaluate the importance of the opinions of significant others to the patient? |
|  |  | **[B]** You told the patient that the ‘dirty spots’ are dental caries and it is an oral disease that requires treatment. | **[B]** The patient felt confused about how the ‘dirty spots’ would be a severe health problem. |  |  |
| 16 | 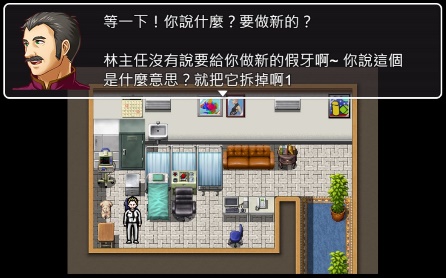A patient asks for ‘removing an old dental bridge’ to treat the teeth inside. He does not realize that the removal of the dental bridge means that the bridge will be broken down and a new one needs to make. | **[A]** You clearly explained to the patient that ‘removing the dental bridge’ is an irreversible step. You asked him to discuss this with his prosthodontist further. | **[A]** The patient misunderstood the treatment, thinking that the bridge can be ‘reused’ in the future. He decided to talk to his prosthodontist first. | • Recognizing the importance of clarifying the ambiguous message (e.g., ‘removal’ of an existing bridge).  • Understanding that patients’ cognition of a procedure may be different from dentists’ perspective, and legal problems may occur due to this diversity | • The patient seemly understands the purpose of the procedure (removal of an existing bridge) but he misunderstood it. How should we help him to clarify his misunderstanding of the procedure?  • When patients surprisingly find themselves getting it wrong, should we keep the original treatment plan going? Or should we have patients reconsider everything before the next step? |
|  |  | **[B]** You clearly explained to the patient that ‘removing the dental bridge’ is an irreversible step. And you just removed it. | **[B]** The patient misunderstood the treatment, thinking that the bridge can be ‘reused’ in the future. However, he still decided to remove it right now. |  |  |
